# The SCImago Journal & Country Rank and the JBRA

**DOI:** 10.5935/1518-0557.20180082

**Published:** 2019

**Authors:** Maria do Carmo Borges de Souza, Paulo Franco Taitson, João Batista Alcântara Oliveira

**Affiliations:** 1 Editor-In-Chief- JBRA Assisted Reproduction; 2 Fertipraxis- Centro de Reprodução Humana- RJ, Brazil; 3 Assistant Editor - JBRA Assisted Reproduction; 4 Discipline of Human Reproduction – Pontifical Catholic University of Minas Gerais (MG), Brazil; 5 Associate Editor - JBRA Assisted Reproduction; 6 Center for Human Reproduction Prof. Franco Jr- Ribeirão Preto (SP), Brazil

The SCImago Journal & Country Rank (SJR) is a publicly available portal that includes journals and scientific indicators from the information contained in the Scopus (Elsevier^®^) database (Bar-Ilan, 2008; Guerrero-Bote & Moya-Anegón, 2012). These indicators can be used to assess and analyze scientific domains. The journals may be compared or analyzed separately. Country rankings can also be compared or analyzed separately. The journals can be grouped by field (27 main subject areas), subject category (313 specific subject categories) or by country. Citation data is drawn from more than 34,100 titles from more than 5,000 international publishers, and performance metrics from 239 countries around the world. The SJCR also enables one to incorporate meaningful daily metrics into one’s web, as a clickable image widget.

We have been in the SJR for nine years, and the great news we want to share with you, members of the SBRA, PRONUCLEO AND REDLARA, authors who help in our growth, and avid readers of the most recent research and information in assisted reproduction: our journal has greatly improved its impact factor and its citation index on the Scopus platform. Following, we share with you all a bit more of what this result means.

The JBRA aims at disseminating scientific knowledge in a broad and unrestricted way, open to ideas and innovations. It is geared towards gynecologists, andrologists, biologists, urologists, nurses and psychologists, among others. It became widely known after fulfilling the necessary requirements to be included in Medline^®^ , the most important existing database, consulted via PUBMED. Initially created to communicate scientific knowledge from the Brazilian Society of Assisted Reproduction (SBRA), it grew by representing PRONUCLEO and the Latin American Network of Assisted Reproduction (REDLARA). It has a quarterly periodicity, and we count on experts from all continents and dimensions of knowledge, a demand from an ever-growing field of publications. The reviewers’ group is extensive, comprising researchers from more than 60 countries. The review of each submission is carried out in a judicious and double blind fashion, and in the shortest time. It is freely accessible to the community through this web link: www.jbra.com.br

As Editors, we happily witness the journal progressing in every year and biennial assessments, showing a 62% increase in the number of papers cited in the last two years, 97% in the number of references; ranking first among the specific journals in human reproduction in Latin America. It is an exponential growth, an advance based on our journal’s internationalization, its popularity among numerous readers outside Latin America, easy and free access via the web, and recently with the publication’s preview, all added to the dedication of those members in our association who daily militate for the scientific quality and reproducible growth in Brazil and Latin America.

The JBRA is doing great, congratulations to all, we continue loyal to our initial mission: open to ideas and suggestions, and promptly available to fulfill its representation role.
